# Pancreas Ligation Device for Distal Pancreatectomy: An Ex Vivo Follow-Up Porcine Study

**DOI:** 10.7759/cureus.44771

**Published:** 2023-09-06

**Authors:** Yuji Kaneda, Yuki Kimura, Akira Saito, Ryusuke Ae, Hiroshi Kawahira, Naohiro Sata

**Affiliations:** 1 Department of Surgery, Division of Gastroenterological, General and Transplant Surgery, Jichi Medical University, Shimotsuke, JPN; 2 Medical Simulation Center, Jichi Medical University, Shimotsuke, JPN; 3 Division of Public Health, Center for Community Medicine, Jichi Medical University, Shimotsuke, JPN

**Keywords:** bioabsorbable, innovation, pressure resistance, porcine study, pancreatic necrosis, pancreatic stump, complication, postoperative pancreatic fistula, distal pancreatectomy, pancreas ligation device

## Abstract

Introduction

Postoperative pancreatic fistula (POPF) is a critical complication occurring with a high incidence after distal pancreatectomy. To minimize the risk of POPF, we developed an innovative pancreas ligation device capable of closing the pancreatic stump without causing traumatic injury to the pancreatic duct and artery. We conducted an *ex vivo* follow-up study to compare the pressure resistance of the pancreas ligation device with that of a regular linear stapler.

Materials and methods

The pancreases were excised from 20 pigs and divided into two groups: ligation group (*n* = 10) and stapler group (*n* = 10). Distal pancreatectomy was performed, and the pancreatic stump was closed using either a pancreas ligation device or a regular linear stapler. The main pancreatic duct was cannulated with a 4-French catheter connected to a cannula and syringe filled with contrast medium. Using fluoroscopy detection, pressure resistance was defined as the maximum pressure without leakage from the pancreatic stump.

Results

No significant differences were found between the two groups regarding sex, age, body weight, or pancreatic thickness. In the ligation group, no leakage was observed at the stump in any pancreas. However, in the stapler group, six of 10 pancreases showed leakage at the staple line or into the parenchyma. Pressure resistance was significantly higher in the ligation group than in the stapler group (median: 42.8 vs. 34.3 mmHg, *P* = 0.023).

Conclusions

These findings suggest the effectiveness of a pancreas ligation device in reducing the incidence of POPF after distal pancreatectomy. Our ligation device is expected to be a useful alternative to a linear stapler for pancreatic stump closure.

## Introduction

Postoperative pancreatic fistula (POPF) is a critical postoperative complication of distal pancreatectomy. POPF can cause fatal secondary complications, such as intra-abdominal hemorrhage, abscess, and sepsis [1-4], resulting in prolonged hospitalization [1,3,5] and increased medical costs [6,7]. Furthermore, POPF is associated with adjuvant chemotherapy failure [8,9], leading to a worsened prognosis in cases of pancreatic cancer [10,11].

Although several new techniques and devices have been introduced for distal pancreatectomy, the incidence of clinically relevant POPF [12] remains high at 15-22% [13-16]. Recently, reinforced staplers have been used for pancreatic stump closure; however, multicenter randomized clinical trials have not demonstrated any advantage of reinforced staplers over regular linear staplers for clinically relevant POPF prevention [17,18]. For these reasons, the optimal closure methods for pancreatic stump to avoid POPF complications after distal pancreatectomy remain controversial [19].

We previously proposed that the pancreas ligation device can potentially prevent POPF after distal pancreatectomy due to its atraumatic ligation approach, with the hypothesis that conventional closure methods for pancreatic stump (e.g., hand-sewn closure, regular linear stapler, and reinforced stapler) cause direct injury to the pancreatic parenchyma and pancreatic duct by staples or needles, thereby increasing the risk of POPF development [20].

To minimize the risk of POPF, we developed an innovative pancreas ligation device capable of closing the pancreatic stump without causing traumatic injury to the pancreatic duct and artery [20]. During the process of improving the device, we modified it from a band type [20] to a front-to-back clamping type, aiming to equalize the ligature pressure and transmit it to the center of the ligature.

To evaluate the effectiveness of the modified pancreas ligation device in reducing the risk of POPF, we conducted an *ex vivo* study comparing the pressure resistance of the main pancreatic duct (MPD) after distal pancreatectomy with a regular linear stapler. This was a follow-up study to verify the findings of the initial pilot *in vivo* study [20].

## Materials and methods

Pancreas ligation device

**Figure 1:**
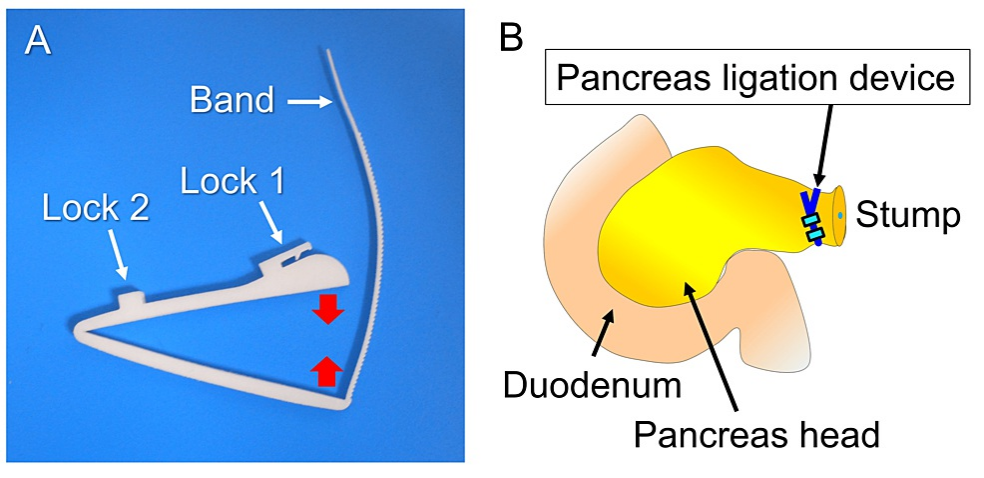
Pancreas ligation device. (A) The ligation device can ligate the pancreas from the front and back and has an adjustable locking system. (B) Image of the ligation device after distal pancreatectomy. The device ligates the pancreatic stump atraumatically. Image credits: Yuji Kaneda

We developed an innovative pancreas ligation device for distal pancreatectomy with technical support from Teijin Medical Technologies Co., Ltd. (Osaka, Japan), made from a bioabsorbable polymer with four specific characteristics (Fig. 1A) [20].

First, the device ligates the pancreas from the front and back without using any directly traumatic components, such as staples and needles (Fig. 1A, 1B). Second, an adjustable locking system controls the ligation force to maintain the arterial blood flow to the pancreatic stump (Fig. 1A). Third, the grooves prevent the device from sliding or displacing the ligature site. Finally, the device is made of a bioabsorbable polymer, leaving behind no foreign bodies. We conducted this *ex vivo* study using a prototype of the device created from non-absorbable plastic with a three-dimensional printer.

*Ex vivo *pancreas and distal pancreatectomy

This study was approved by the Animal Experiment Committee of our institution (approval no. 18035-02), and all animals were managed according to the ethical regulations for animal studies. Twenty pigs (10 female and 10 castrated male Landrace pigs) were purchased from Sanesu Breeding Co., Ltd. (Funabashi, Japan). The pancreases were individually excised under general anesthesia and thereafter were wrapped, stored at -25 °C, and finally were divided into two groups: ligation group (*n *= 10) and stapler group (*n* = 10).

**Figure 2:**
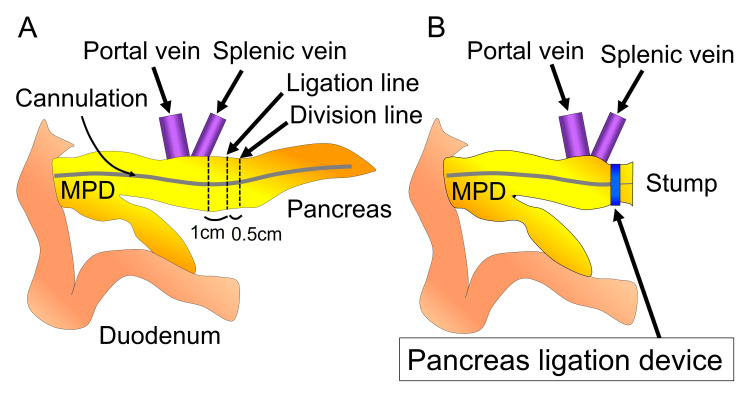
Schematic illustration of porcine distal pancreatectomy. (A) Ligation and division line of the pancreas. The ligation and division lines are set at 1 cm and 1.5 cm to the left of the confluence of the splenic vein. Pancreases in the stapler group were divided across the division line, (B) while those in the ligation group were first ligated across the ligation line and then divided across the division line. MPD, main pancreatic duct Image credits: Yuji Kaneda

**Figure 3:**
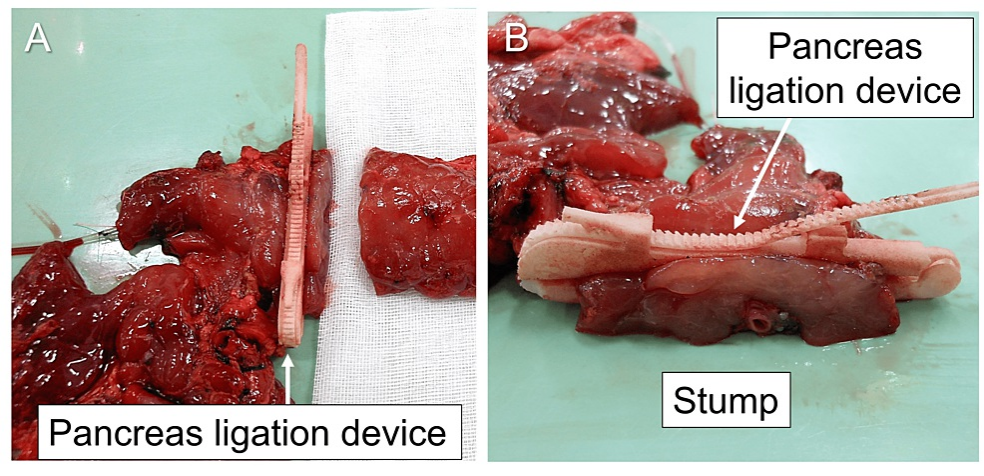
Pancreatic stump closure with the pancreas ligation device. (A) The pancreas is divided with Metzenbaum scissors. (B) The pancreatic stump is ligated with the pancreas ligation device.

Distal pancreatectomy was performed with defrosted pancreases, and the pancreatic stump was closed using a pancreas ligation device and regular linear stapler in the ligation and stapler groups, respectively. In the ligation group, we ligated the pancreas at 1 cm to the left of the confluence of the splenic vein and divided the pancreas with Metzenbaum scissors at 0.5 cm from the ligation line (Fig. 2A, 2B, 3A, 3B). The ligation force of the device was to the locking limit (Fig. 1A, 3B).

**Figure 4:**
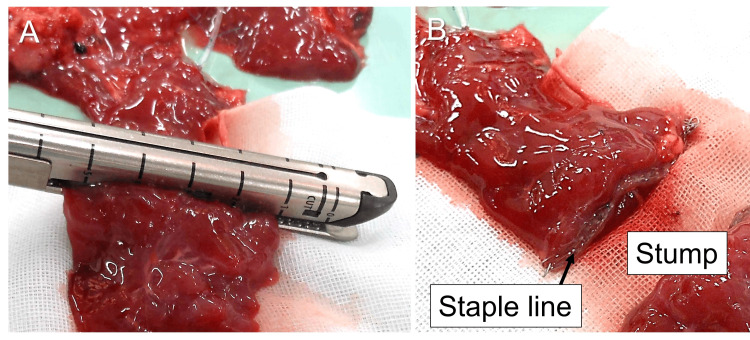
Pancreatic stump closure with a linear stapler. (A) The pancreas is divided with a linear stapler. (B) The pancreatic stump is closed with staples.

In the stapler group, we divided the pancreas with a linear stapler (Endo GIA^TM^ Black Reload with Tri-Staple^TM^ Technology 60 mm; Covidien Japan, Inc., Japan) at 1.5 cm to the left of the splenic vein confluence (Fig. 2A, 4A, 4B).

Pressure resistance test

**Figure 5:**
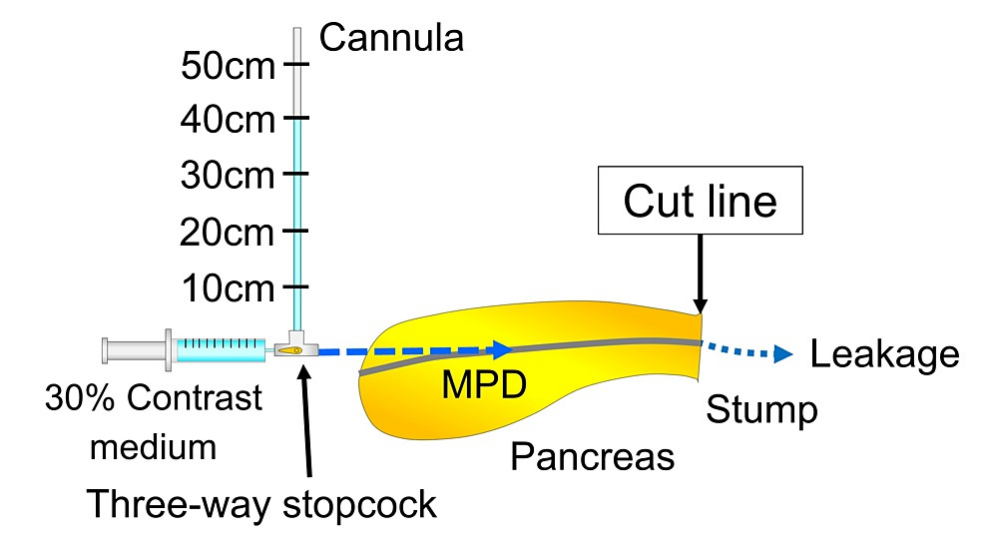
Schematic illustration of the pressure resistance test. The MPD is cannulated through the duodenal lobe of the pancreas. The catheter is connected to the cannula and syringe by a three-way stopcock. The pancreas is cut along the cut line. Leakage from the pancreatic stump is detected by X-ray fluoroscopy. MPD, main pancreatic duct. Image credits: Yuji Kaneda.

The MPD pressure after distal pancreatectomy was compared between the ligation and stapler groups. The MPD was cannulated with a 4-French catheter placed through the duodenal lobe of the pancreas, which corresponds to the head of the pancreas in humans (Fig. 5).

The catheter was then connected to a cannula marked from 5 to 50 cm (at 5-cm intervals, 10 steps) and a syringe filled with 30% contrast medium. The cannula was filled with a contrast medium, and the MPD pressure was increased from 5 to 50 cm (at 5-cm intervals, 10 steps). This operation increased the pressure from 4.3 to 42.8 mmHg in 4.3-mmHg increments. After opening the stopcock, the MPD pressure was maintained for five seconds by holding the stopcock at each level. Contrast leakage from the pancreatic stump or staple line was detected using fluoroscopy. We defined the MPD pressure resistance as the maximum pressure without leakage.

Statistical analyses

The numerical variables are presented as the median (minimum to maximum). The Mann-Whitney U test was performed to compare the MPD pressure resistance values. The significance threshold was set at *P* < 0.05. All analyses were performed using the IBM SPSS Statistics software program for Windows, version 29 (released 2022, IBM Corp., Armonk, NY, USA).

## Results

No significant differences were found between the ligation and stapler groups in the sex (castrated male/female ratio: 6/4 vs. 4/6), age (94 (79 to 118) vs. 88 (76 to 110) days old), body weight (34.0 (27.8 to 40.2) vs. 33.8 (30.0 to 40.5) kg), or thickness of the pancreas (8 (6 to 12) vs. 10 (5 to 11) mm).

**Figure 6:**
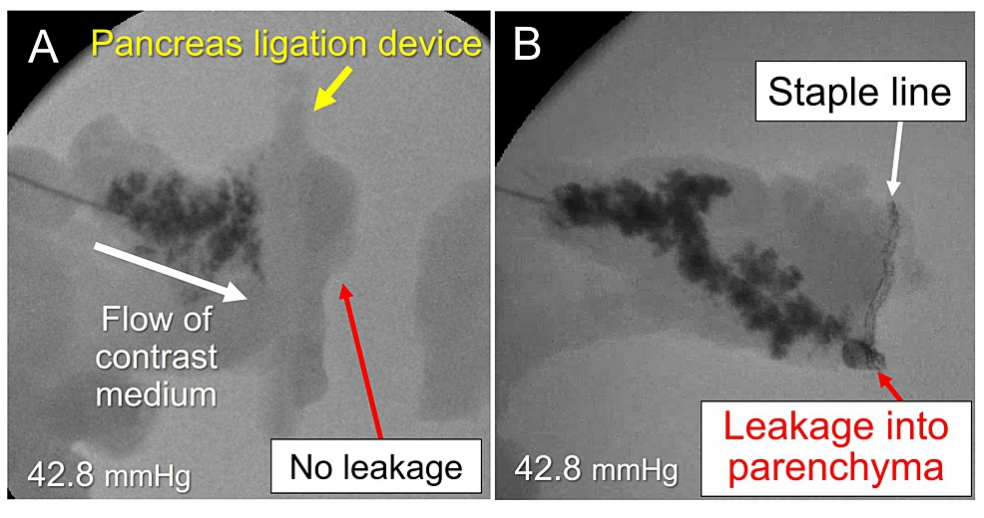
Fluoroscopic imaging during the pressure resistance test. (A) Fluoroscopic view with the pancreas ligation device. The device stops the flow of contrast medium, with no leakage detected from the pancreatic stump. (B) Fluoroscopic view after closure with the linear stapler. Leakage into the parenchyma is detected.

**Figure 7:**
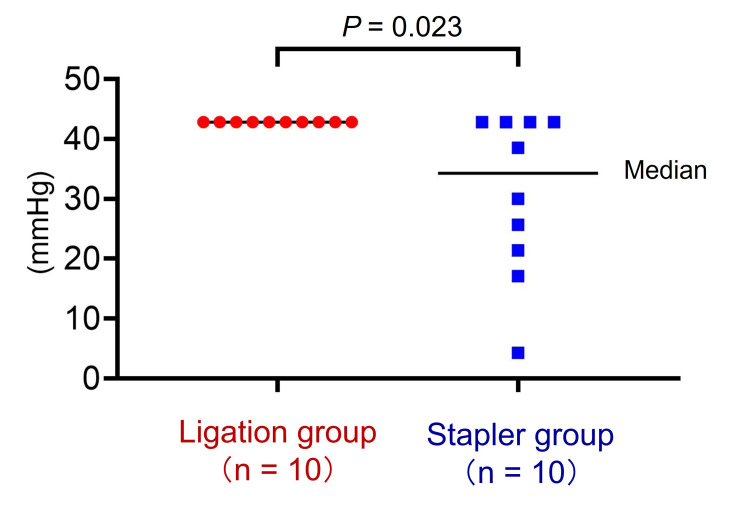
Main pancreatic duct pressure resistance test results. The pressure resistance is significantly higher in the ligation group than in the stapler group (*P* = 0.023).

No leakage was observed at the stump in the pancreas in the ligation group (Fig. 6A). However, in the stapler group, six of 10 pancreas showed leakage at the staple line or into the parenchyma (Fig. 6B). The MPD pressure resistance was significantly higher in the ligation group than in the stapler group (median: 42.8 vs. 34.3 mmHg, *P* = 0.023) (Fig. 7).

## Discussion

In a previous pilot study [20], we introduced an innovative pancreas ligation device potentially able to prevent POPF after distal pancreatectomy by atraumatic ligation. The device ligates the pancreatic stump while maintaining the arterial blood flow and limiting pancreatic necrosis. After modification of the ligation device, the present *ex vivo* follow-up study was conducted to evaluate the effectiveness of the improved ligation device by comparing the MPD pressure resistance with the improved ligation device against that with a regular linear stapler. The present study highlights an important finding. That is, compared to the regular linear stapler closure, this new device adequately ligated the pancreatic stump with a significantly higher MPD pressure resistance, thereby reducing the risk of POPF.

The normal MPD pressure among 20 healthy humans was estimated as a mean of 16.2 mmHg with a range of 3-31 mmHg [21,22]. A previous *ex vivo* study reported that the mean pressure associated with leakage after distal pancreatectomy was 13.7 mmHg when using 2.3-mm staples for stump closure [23]. These findings suggested that pancreatic leakage might occur within the normal MPD pressure in humans after distal pancreatectomy when using a routine linear stapler. Based on these reports, a pancreatic stump should be higher than 13.7 mmHg (the stapler's mean pressure) and ideally higher than 31 mmHg (the upper limit of pancreatic duct pressure in healthy humans) as described in the above previous studies. In the present study, all pancreases in the ligation group demonstrated no leakage at a median MPD pressure of 42.8 mmHg, which was significantly higher than that in the stapler group. Thus, our device can close the pancreatic stump with sufficient MPD pressure resistance to reduce the risk of POPF.

Surgical staplers are typically used to close the pancreatic stump in distal pancreatectomy. A multicenter randomized controlled trial (DISPACT trial) comparing stapler and hand-sewn closure of the pancreatic stump previously determined no significant difference in the occurrence of POPF with subsequent death after distal pancreatectomy until postoperative day seven [4]. Several new techniques and devices have been introduced to minimize POPF complications after distal pancreatectomy, such as pancreaticojejunostomy and reinforced staplers. However, a multicenter randomized controlled trial showed that pancreaticojejunostomy did not significantly reduce the occurrence of POPF after distal pancreatectomy compared to conventional surgical stapler closure [24]. Although some studies have reported the superiority of reinforced staplers over regular stapler closure [25,26], the occurrence of clinically relevant POPF has remained high at 5-16% [25-27]. In multicenter randomized clinical trials, reinforced staplers did not show any advantage over regular staplers for clinically relevant POPF prevention [17,18]. These conventional methods cannot close the pancreatic stump without penetrating the pancreatic parenchyma with staples or needles. We hypothesized that such injuries would lead to pancreatic duct and small artery injury at the pancreatic stump.

Our previous report [20] showed that ischemia of the pancreatic stump is associated with pancreatic necrosis. Nagakawa et al. [23] reported that hand-sewn closure caused pancreatic necrosis and regenerated the pancreatic ducts in mongrel dogs, potentially leading to POPF. These reports suggest that maintaining the arterial blood flow to the pancreatic stump may reduce the risk of necrosis and incidence of POPF. Yamashita et al. [28] developed a clip-type device to reduce injury to the pancreatic parenchyma during stump closure. This clip has a space between the upper and lower jaws to avoid causing direct injury to the pancreatic parenchyma. However, the clip may not control the ligation force, thereby less controlling the arterial blood flow to the pancreatic stump. An ideal device for pancreatic stump closure can minimize the risk of pancreatic duct and artery injury during ligation while maintaining the arterial blood flow to the pancreatic stump [20]. Our new ligation device addresses these previous issues and may also reduce the incidence of POPF.

The present study has some limitations. First, a porcine pancreas was used as a substitute for the human pancreas to assess leak pressure. Our findings may therefore not be directly generalizable to human clinical practice. However, the porcine pancreas has soft parenchyma, similar to the human pancreas, and has been used in studies of distal pancreatectomy [29,30]. Second, the study did not assess any ischemic changes at the pancreatic stump or ligation site because of the *ex vivo* experimental study design. The ligation pressure in this study might affect arterial blood flow reduction to the pancreatic stump. Although the band-type ligation device [20] could preserve arterial blood flow to the pancreatic stump and prevent POPF, future *in vivo* studies using a front-to-back clamping-type device are warranted to confirm the preservation of arterial blood flow.

## Conclusions

The present study demonstrates the effectiveness of an innovative pancreas ligation device in reducing the incidence of POPF after distal pancreatectomy. Compared to the regular linear stapler closure, the device ligated the pancreatic stump with adequate pressure to avoid leakage. Our ligation device is expected to be a useful alternative to a linear stapler for pancreatic stump closure.
